# Between Limb Muscle Co-activation Patterns in the Paretic Arm During Non-paretic Arm Tasks in Hemiparetic Cerebral Palsy

**DOI:** 10.3389/fnins.2021.666697

**Published:** 2021-07-29

**Authors:** Nayo M. Hill, Theresa Sukal-Moulton, Julius P. A. Dewald

**Affiliations:** ^1^Department of Physical Therapy and Human Movement Sciences, Northwestern University, Chicago, IL, United States; ^2^Department of Biomedical Engineering, Northwestern University, Evanston, IL, United States; ^3^Department of Pediatrics, Northwestern University, Chicago, IL, United States; ^4^Department of Physical Medicine and Rehabilitation, Northwestern University, Chicago, IL, United States

**Keywords:** mirror movements, muscle co-activation, cerebral palsy, childhood hemiplegia, perinatal stroke

## Abstract

Tasks of daily life require the independent use of the arms and hands. Individuals with hemiparetic cerebral palsy (HCP) often experience difficulty with fine motor tasks demonstrating mirrored movements between the arms. In this study, bilateral muscle activations were quantified during single arm isometric maximum efforts and submaximal reaching tasks. The magnitude and direction of mirrored activation was examined in 14 individuals with HCP and 9 age-matched controls. Participants generated maximum voluntary torques (MVTs) in five different directions and completed ballistic reaches while producing up to 80% of shoulder abduction MVT. Electromyography (EMG) signals were recorded from six upper extremity muscles bilaterally. Participants with HCP demonstrated more mirrored activation when volitionally contracting the non-paretic (NP) arm than the paretic arm (*F* = 83.543, *p* < 0.001) in isometric efforts. Increased EMG activation during reach acceleration resulted in a larger increase in rest arm co-activation when reaching with the NP arm compared to the paretic arm in the HCP group (*t* = 8.425, *p* < 0.001). Mirrored activation is more pronounced when driving the NP arm and scales with effort level. This directionality of mirroring is indicative of the use of ipsilaterally terminating projections of the corticospinal tract (CST) originating in the non-lesioned hemisphere. Peripheral measures of muscle activation provide insight into the descending pathways available for control of the upper extremity after early unilateral brain injury.

## Introduction

Mirror movements in the arm are defined as involuntary mimicking of voluntary movement of the contralateral arm (Green, 1967; Woods and Teuber, 1978; Nelles et al., 1998; Riddell et al., 2019). In the immature nervous system, the presence of primarily bilateral corticospinal projections between the cortex and spinal motor circuits lends itself to mirror movements (Eyre et al., 2001). Independent control of the arm is refined and mirroring diminishes with age (Woods and Teuber, 1978; Koerte et al., 2010) as typical nervous system maturation prunes descending motor pathways. This process results in contralateral connections of over 80% of the corticospinal tract (CST) and loss of ipsilaterally terminating projections. A principle exception to this typical progression is a unilateral lesion to the developing brain such as a perinatal stroke resulting in hemiparetic cerebral palsy (HCP). The alternative connectivity patterns of descending pathways from the interruption to the typical development of the CST could result in a persistence of mirror movements (Eyre et al., 2001; Staudt, 2010; Jaspers et al., 2015). Indeed, mirror movements were documented in 1978 during a supination task in children with hemiplegia, most prominently seen in participants with lesions occurring before 1 year of age (Woods and Teuber, 1978).

Mirror movements have continued to be investigated using observational rating scales scoring their presence/absence and frequency during different tasks (Kuhtz-Buschbeck et al., 2000; Riddell et al., 2019). Assessment of mirror movements has expanded beyond observational rating scales by using grasp force measurement devices that are suitable for a clinical setting (Jaspers et al., 2018; Zielinski et al., 2018). Kuhtz-Buschbeck et al. (2000) classified the presence of mirror movements qualitatively and quantitatively during both unimanual and bimanual grasp force tasks. Their key finding was that participants with HCP were able to suppress mirror movements when cued in an experimental setting, but the presence of significant mirroring was related to a reduction in non-dominant (ND) hand use during functional bimanual tasks (Kuhtz-Buschbeck et al., 2000), suggesting that suppression of mirror movements is only possible in specific scenarios. In a similar manner, Adler et al. (2015) found a negative impact of mirror movements on complex bimanual tasks. These findings highlight the functional implications of mirror movements motivating the need for continued exploration and understanding of the underlying neural mechanism of this motor observation.

Weinstein et al. (2018) present a case series of individuals with unilateral CP using a multimodal approach of transcranial magnetic stimulation (TMS), electromyography (EMG), magnetic resonance imaging (MRI), and electroencephalography (EEG) to demonstrate the benefits of using several different neuroimaging methods to assess mirror movements. Other researchers have used a smaller number of modalities to quantify specific contributing components to the phenomenon of mirror movements. TMS has been used to describe an abnormal ipsilateral wiring pattern of the CST implicated in the control of the paretic hand (Eyre et al., 2007; Staudt, 2010; Mailleux et al., 2020). Ipsilateral wiring is defined when motor evoked potentials (MEPs) are detected in the paretic hand during stimulation of the non-lesioned cortical hemisphere. Carr et al. (1993) provide two hypotheses for the neural mechanism of the ipsilateral wiring patterns detected in their test cohort: (1) the axons from the non-lesioned cortex may have branched to bilateral motor neuron pools in the spinal cord; or (2) axons descend bilaterally from the non-lesioned cortex but are separate, un-branched projections terminating bilaterally in the spinal cord (Carr, 1996). While connectivity of descending pathways has been established with TMS, the link to the behavioral presentation and directionality of mirroring has not been as well-characterized. TMS is robust in determining the existence of a connection between the non-lesioned cortex and the intrinsic muscles in the paretic hand. However, TMS is limited in determining whether that pathway is a mono-synaptic crossed CST projection, a lower resolution mono-synaptic ipsilateral CST projection, an oligosynaptic projection through the brainstem, or some combination of these connections (Carr et al., 1993; Staudt et al., 2002).

Peripheral measures of muscle activity can be used to gain insight on the functional output of the nervous system. EMG has been used in HCP to characterize within limb muscle synergy patterns (Tang et al., 2017), abnormal muscle coupling patterns (Sukal-Moulton et al., 2014), and between limb mirrored muscle activations (Green, 1967; Sukal-Moulton et al., 2013; Riddell et al., 2019). However, previous studies have either focused on muscles crossing a single joint, captured the timing of mirrored activations but not the amplitude, or only investigated mirroring in one direction (Green, 1967; Sukal-Moulton et al., 2013).

In the present study, we use volitional EMG during maximal isometric and submaximal dynamic efforts to characterize the magnitude and direction of mirrored muscle activation in the whole arm and explore the hypotheses for neural connectivity put forth by Carr et al. (1993). Four possible mirroring scenarios were evaluated: (1) paretic arm mirrors when non-paretic (NP) arm is active (P_Mirror; Carr hypothesis 1; Carr et al., 1993), (2) NP arm mirrors when paretic arm is active (N_Mirror; Carr hypothesis 2; Carr et al., 1993), (3) both arms mirror (B_Mirror; combination of mechanisms), or (4) neither arm mirrors (X_Mirror; independent pathways). Additionally, our methods allow us to determine whether there are differences in amount of mirroring within the limb. While mirror movements, or associated reactions (Brunnstrom, 1970), observed in adults following stroke are thought to be mediated by pathways synapsing in the brainstem (Ejaz et al., 2018), the absence of abnormal flexion synergies detected in individuals with HCP indicates that use of a brainstem pathway is unlikely to significantly influence arm control in this population (Hill and Dewald, 2020). We hypothesized that individuals with HCP would show mirroring in the paretic arm primarily when activating the NP arm and not the reverse (P_Mirror) explained by maintained ipsilateral projections branching from the CST descending from the non-lesioned cortical hemisphere (Staudt et al., 2002; Alawieh et al., 2017). Furthermore, since the CST is most uniquely involved in control of the distal part of the extremity (Lawrence and Kuypers, 1968; Porter and Lemon, 1993; ten Donkelaar et al., 2004), we hypothesize that there might be increased expression of mirroring distally vs. proximally.

## Materials and Methods

### Participants

Individuals with HCP were identified through the Cerebral Palsy Research Registry (Hurley et al., 2011), local clinics, and parent support groups. In brief, participants were; (1) at least 6 years of age at time of testing; and (2) had a diagnosis of CP with unilateral motor impairment of the upper extremity; full criteria have been reported previously (Hill and Dewald, 2020). A cohort of age-matched controls without known neurological impairment (typical development; TD) was recruited for comparison. This experimental protocol was approved by the Institutional Review Board of Northwestern University. All participants were minors; parents provided informed consent prior to participation and participants provided assent.

Grip strength was recorded with the participant sitting with the shoulder at 0° of abduction and elbow at 90° of flexion. A ratio was calculated of the paretic to NP hand or ND to dominant hand (Jamar Hand Dynamometer, B&L Engineering, Tustin, CA, United States). For the cohort with HCP, a number of clinical assessments were used to classify function and impairment including: the Gross Motor Function Classification Scale (GMFCS) (Palisano et al., 1997; Rosenbaum et al., 2008), the Manual Abilities Classification Scale (MACS) (Eliasson et al., 2006), the Test of Arm Selective Control (TASC) (Krosschell et al., 2015; Sukal-Moulton et al., 2018), the Fugl-Meyer Assessment-Upper Extremity (FMA-UE) (Fugl-Meyer et al., 1975; Fasoli et al., 2009), and the ABILHAND-Kids (ABL-H) (Penta et al., 2001; Arnould et al., 2004).

### Experimental Setup and Protocol

All experimental conditions were tested one arm at a time with the first arm tested randomized for each participant. The active arm is referred to as the *“Active_Isometric Arm”* for isometric tasks and *“Active_Reaching Arm”* for dynamic tasks. The contralateral arm was cued to rest and is referred to as the *“Rest Arm.”* To determine the presence/absence of mirrored muscle activation and directionality when executing a single arm task, participants completed the following set of static and dynamic tasks in each arm.

#### Maximum Isometric Efforts

Participants were seated in a Biodex experimental chair (Biodex Medical Systems, Inc., Shirley, NY, United States) with chest and lap straps to minimize movement of the trunk. Their skin was cleaned to remove dirt and oils to decrease skin impedance. Active differential surface electrodes with 1-cm inter-electrode distance (Delsys, 16-channel Bagnoli EMG System, Boston, MA, United States; 1,000×gain, 20 and 450 Hz bandpass filter) were placed by a licensed physical therapist on the following six upper extremity muscles on each arm: biceps brachii (BIC; elbow flexor), lateral head of the triceps brachii (TRI; elbow extensor), intermediate deltoid (IDL; shoulder abductor), brachioradialis (BRD; elbow flexor), extrinsic wrist/finger flexors (WFL; flexor carpi radialis and flexor digitorum profundus), and extrinsic wrist/finger extensors (WEX; extensor carpi radialis, extensor digitorum communis). After electrode placement, the forearm of the arm randomized as the first *Active_Isometric Arm* was casted with a participant specific cast from just distal to the epicondyles of the humerus to the fingertips (Hill and Dewald, 2020). This arm was positioned at 85° shoulder abduction, 40° shoulder horizontal flexion, and 90° elbow extension as previously described (Hill and Dewald, 2020). Participants were cued to produce and hold a 5 s maximum effort in five joint torque directions: shoulder abduction, elbow flexion, elbow extension, wrist flexion, and wrist extension. No visual feedback of muscle contraction amplitudes was provided. To elicit the maximum voluntary contraction, at least three trials were recorded for each muscle with consistent performance (within 10% of each other) with the last trial not being the maximum. Breaks of a minimum of 10 s between contraction trials were given to prevent muscle fatigue. Three participants were not casted and instead isometric maximal contraction efforts were completed with manual resistance provided by a research physical therapist. The contralateral arm, termed *Rest Arm*, was cued to relax during the *Active_Isometric Arm* efforts. Muscle activations were recorded and saved through a data acquisition device (NI-DAQ USB-6225; National Instruments, Austin, TX, United States) at 1,000 Hz using customized MATLAB software (Mathworks, Inc., Natick, MA, United States).

#### Submaximal Reaching Efforts

Participants also completed a set of reaching tasks using a haptic robotic device called the Arm Coordination Training 3D (ACT-3D) using a protocol described previously (Hill and Dewald, 2020). The ACT-3D is a device that includes the admittance controlled HapticMASTER (Moog-FCS BV, The Netherlands), a six-degree of freedom load cell end effector (JR3, Woodland, CA, United States; model no. 51E20A4), an instrumented gimbal to record joint angles, and a Biodex experimental chair (Biodex Medical Systems, Shirley, NY, United States), as described previously (Sukal et al., 2007; Ellis et al., 2008, 2016; Hill and Dewald, 2020). During the submaximal reaching tasks, the *Active_Reaching Arm* was rigidly attached to the ACT-3D and participants were cued to complete a set of ballistic forward reaching tasks with shoulder abduction load modulated by a percentage of shoulder abduction maximum voluntary torque (MVT). For all but the table supported condition, participants were instructed to lift the *Active_Reaching Arm* up off of the virtual table, hold for 2 s, and maintain the lift for the duration of the reach. Participants completed reaching with the arm fully supported on the table and generating up to 80% of shoulder abduction MVT. EMG from the same six muscles as in the maximum effort tasks were collected bilaterally during the ballistic reaching tasks.

### Data Analysis

All analysis was performed in MATLAB. Raw EMG signals for each muscle were full-wave rectified, baseline corrected, and manually inspected for artifacts. Artifacts were removed prior to smoothing of data with a moving average filter (250 ms window). Signals for each muscle were normalized to the maximum value recorded for that muscle across all maximal and submaximal tasks for that participant. This normalization strategy was selected to capture the highest capacity of the muscle, regardless of whether it was a voluntary effort or an involuntary co-activation.

#### Mirroring During Isometric Maximum Efforts

The timepoint of the maximum volitional contraction during the static contraction efforts was identified and EMG activity for the cued agonist and the same muscle on the opposite arm was recorded. To quantify the presence of between limb co-activation of the same muscle, a mirrored ratio was calculated for each muscle during volitional contractions using Equation 1. In this equation for a particular muscle, IsometricArm_Muscle_*EMG*_ is the normalized EMG activity for the muscle in the *Active_Isometric Arm* and RestArm_Muscle_*EMG*_ is the normalized EMG activity for the muscle in the *Rest Arm*. The ratio ranges from 0 to 0.5 where a value of 0 indicates no mirroring—only the isometric arm muscle is active, and a value of 0.5 indicates strong mirroring—the muscle is equally active in both arms.

$$M\,i\,r\,r\,o\,r\,e\,d\,R\,a\,t\,i\,o_{M\,u\,s\,c\,l\,e}=\frac{R\,e\,s\,t\,A\,r\,m\,\_\,M\,u\,s\,c\,l\,e_{E\,M\,G}}{I\,s\,o\,m\,e\,t\,r\,i\,c\,A\,r\,m\,\_\,M\,u\,s\,c\,l\,e_{E\,M\,G}+R\,e\,s\,t\,A\,r\,m\,\_\,M\,u\,s\,c\,l\,e_{E\,M\,G}}$$

#### Bilateral Activation During Submaximal Reaching Tasks

**FIGURE 1:**
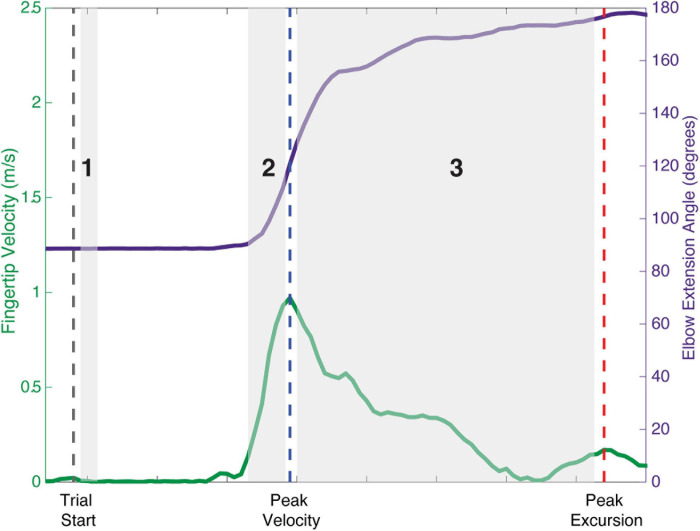
Schematic of analysis timepoints during reaching tasks with movement onset, peak velocity, and peak excursion indicated. Timepoint 1: 25 ms window during static hold before the reach. Timepoint 2: 100 ms window prior to peak velocity representing the acceleration phase of the reach. Timepoint 3: deceleration phase of the reach between peak velocity and peak excursion.

To evaluate the effect of modulating descending input to the shoulder abductors on the presence of bilateral muscle activity in all of the muscles of the arm, a total arm EMG summation was calculated for the *Active_Reaching Arm* and the *Rest Arm* for each load level of the reaching task. Three timepoints were evaluated as can be seen in Figure 1: (1) 25 ms window during static hold (before reach), (2) 100 ms window prior to peak velocity (acceleration of reach), and (3) between peak velocity and peak excursion (deceleration of reach). Timepoint 1 captures the muscle activation corresponding to shoulder abduction prior to integration of sensory feedback. Timepoint 2 captures muscle activation contributing to the combination of shoulder abduction, shoulder flexion, and elbow extension. Timepoint 3 captures the muscle activation contributing to the arm slowing down.

### Statistical Analysis

Statistical analysis was completed using SPSS software (version 26, SPSS Inc., Chicago, IL, United States). A value of *p* < 0.05 was considered statistically significant for all tests. Q-Q plots were used to inspect data distributions and detect outliers. *T*-tests were used to determine whether there were differences in age or grip ratio between TD and HCP groups and Fisher’s Exact tests were used to determine if there were differences in sex and writing hand.

To evaluate whether the HCP and TD groups mirrored differently during isometric efforts, mirrored ratios were analyzed with a generalized linear mixed effects model for each arm with factors of group (TD or HCP) and muscle (IDL, BIC, BRD, TRI, WFL, and WEX), and a random factor of participant. To evaluate whether there was a directionality of mirroring within the TD or HCP group, mirrored ratios were analyzed with a generalized linear mixed effects model with factors test arm (Dominant/NP or ND/Paretic) and muscle (IDL, BIC, BRD, TRI, WFL, and WEX), and a random factor of participant. For each model, *post hoc* comparisons with Bonferroni corrections for multiple comparisons were made for significant main effects. To evaluate the effect of muscle activation in the *Active_Reaching Arm* on muscle activation in the *Rest Arm*, EMG summations were compared in a linear mixed effects model with fixed factor of group_arm (TD_Dominant, TD_NonDominant, HCP_NonParetic, or HCP_Paretic), random factor of participant, and a covariate of *Active_Reaching Arm* EMG. For each group_arm combination, the parameter estimates of the group_arm by *Active_Reaching Arm* EMG interaction were compared using *t*-tests to detect differences in the relationship (slope) between activation in the *Active_Reaching Arm* and the *Rest Arm*.

## Results

Fourteen individuals with HCP (12.08 ± 3.69 years old; 9 male) and nine individuals with no history of neurological impairment (TD; 10.93 ± 3.86 years old; 5 male) participated in this study (Table 1).

**TABLE 1:** Participant characteristics by group.

	**TD (*n* = 9)**	**HCP (*n* = 14)**
Age, mean (SD), years	10.93 (3.87)	12.07 (3.69)
Sex, n		
Male	5	9
Female	4	5
Grip ratio, mean (SD)*	0.931 (0.11)	0.459 (0.32)
Dominant/non-paretic arm, n		
Right	8	4
Left	1	10
GMFCS, n		
I	NA	7
II	NA	6
III	NA	1
MACS, n		
I	NA	5
II	NA	7
III	NA	2
FMA-UE, mean (SD), x/66	NA	42.86 (14.13)
TASC-P, mean (SD), x/16	NA	7.812 (2.79)
TASC-NP, mean (SD), x/16	NA	13.09 (2.55)
ABL-H, mean (SD), logit	NA	3.58 (1.88)

*Grip ratio calculated as grip strength in non-dominant/dominant or paretic/non-paretic. ABL-H, ABILHAND-Kids; FMA-UE, Fugl-Meyer Assessment Upper Extremity; GMFCS, Gross Motor Function Classification System; HCP, hemiparetic cerebral palsy; MACS, Manual Abilities Classification Scale; SD, standard deviation; TASC-NP, Test of Arm Selective Control non-paretic arm; TASC-P, Test of Arm Selective Control paretic arm; TD, typical development.

### Comparison of Participant Characteristics and Functional Scores

Normality of age and grip ratio distributions was confirmed with Shapiro–Wilk tests. There was not a significant difference in age (*t*_21_ = −0.712, *p* = 0.484, *g* = −0.293) or sex (*p* = 1.00, phi-coefficient = −0.087) between the test groups. More individuals in the HCP group wrote with their left hand compared to the TD group (*p* = 0.009, phi-coefficient = 0.589). The HCP group had significantly lower grip strength ratios (*t*_17_ = 5.139, *p* < 0.001, *g* = 1.765) indicating a weaker paretic hand.

### Mirroring During Isometric Maximum Efforts

**FIGURE 2:**
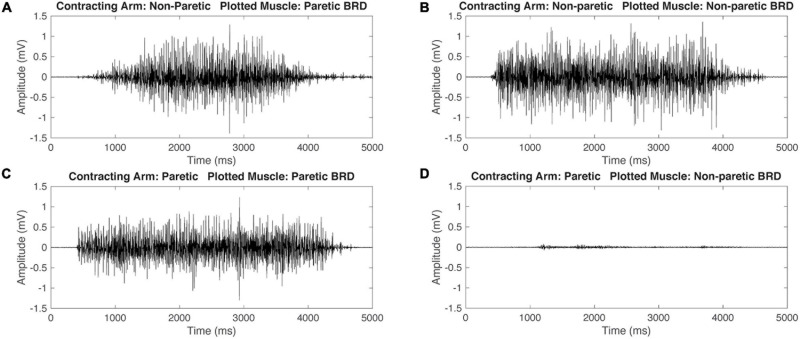
Representative raw electromyography (EMG) signals during isometric maximum contractions of the brachioradialis (BRD) for a participant with hemiparetic cerebral palsy (HCP). When contracting the non-paretic (NP) arm, both the paretic and non-paretic BRD are activating simultaneously **(A,B)**. When contracting the paretic arm, the paretic arm BRD is activating and the NP arm has minimal activation **(C,D)**. This demonstrates mirrored activation during NP arm contraction but not during paretic arm contraction.

**FIGURE 3:**
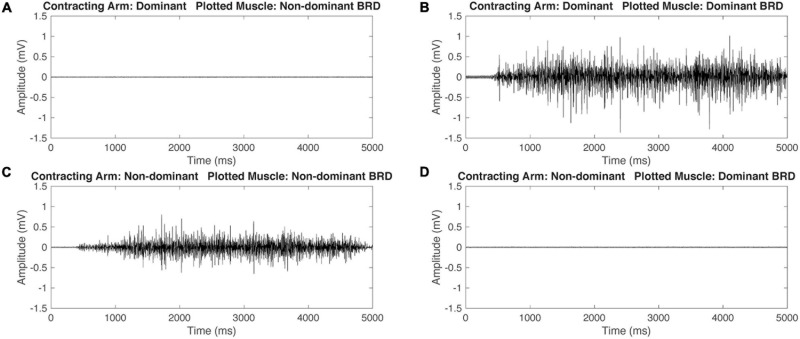
Representative raw electromyography (EMG) signals during isometric maximum contractions of the BRD for a participant with typical development (TD). When contracting the dominant arm, the non-dominant (ND) BRD is quiet **(A)** and the dominant arm BRD is activating **(B)**. Similarly, when contracting the ND arm, the ND arm BRD is activating **(C)** and the dominant arm BRD is quiet **(D)**. This demonstrates that the participant with TD is able to independently activate the BRD during maximal isometric tasks.

Representative EMG data for one participant with HCP and one participant with TD during isometric maximum efforts can be seen in Figures 2, 3. Mirrored ratios were transformed using natural logs prior to statistical analysis to satisfy assumptions of normality. All statistical results presented are based on the transformed data while figures present untransformed data. When contracting the dominant/NP arm, there was a significant main effect of group [*F*_(__1_, _126__)_ = 22.655, *p* < 0.001], muscle [*F*_(__5_, _126__)_ = 4.370, *p* = 0.001], and the interaction of group by muscle [*F*_(__5_, _126__)_ = 6.812, *p* < 0.001] with the HCP group demonstrating larger mirrored ratios for all muscles. *Post hoc* pairwise comparisons revealed a significant difference in mirrored ratios between TD and HCP for BIC (higher in HCP; *t*_126_ = −5.619, *p* < 0.001, *g* = −2.188), WFL (higher in HCP, *t*_126_ = −5.060, *p* < 0.001, *g* = −2.480), WEX (higher in HCP; *t*_126_ = −2.187, *p* = 0.031, *g* = −0.905), and BRD (higher in HCP; *t*_126_ = −4.830, *p* < 0.001, *g* = −1.576) but not TRI (*t*_126_ = −1.270, *p* = 0.206) or IDL (*t*_126_ = −1.231, *p* = 0.221). Additionally, there was a significant difference in mirrored ratio between BIC and WEX (higher in WEX; *t*_126_ = −2.981, *p* < 0.041, *g* = −0.019), IDL and WFL (higher in IDL; *t*_126_ = 3.062, *p* = 0.038, *g* = 0.031), WFL and WEX (higher in WEX; *t*_126_ = −3.489, *p* = 0.010, *g* = −0.033), and BRD and WEX (higher in WEX; *t*_126_ = −3.059, *p* = 0.038, *g* = −0.024). When contracting the ND/paretic arm, there was a significant main effect of muscle [*F*_(__5_, _126__)_ = 6.843, *p* < 0.001] but not group [*F*_(__1_, _126__)_ = 0.124, *p* = 0.725] or the interaction of group by muscle [*F*_(__5_, _126__)_ = 1.904, *p* = 0.098]. *Post hoc* pairwise comparisons revealed a significant difference in mirrored ratio between BIC and IDL (higher in IDL; *t*_126_ = −4.913, *p* < 0.001, *g* = −0.109), BIC and WEX (higher in WEX; *t*_126_ = −4.312, *p* = 0.000, *g* = −0.099), IDL and BRD (higher in IDL; *t*_126_ = 3.714, *p* = 0.004, *g* = 0.097), and BRD and WEX (higher in WEX; *t*_126_ = −3.113, *p* = 0.028, *g* = −0.086). These results highlight a difference in mirroring between TD and HCP groups when completing the volitional task on the dominant/NP arm but not a difference between groups when completing the same task with the ND/paretic arm.

**FIGURE 4:**
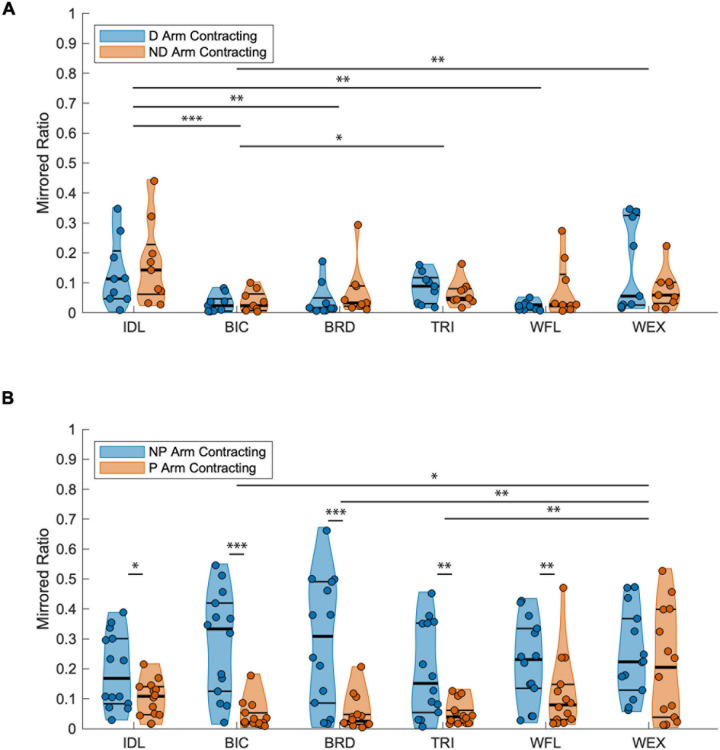
Mirrored ratios by group. Thick horizontal black lines on violin plots indicate group median and thin black lines indicate first and third quartiles. **(A)** TD group; A significant main effect of muscle was found with the IDL being significantly higher than the BIC, BRD, and WFL, the TRI higher than BIC, and the WEX higher than BIC for both the dominant and ND arms as indicated by the horizontal lines above muscle groups. **(B)** HCP group; A significant main effect of both test arm and muscle were found. Pairwise comparisons demonstrated increased mirrored ratios when contracting the NP arm in all muscles except the WEX demonstrating a unilateral directionality of mirroring for the majority of upper extremity muscles. Significance symbols above horizontal lines indicate differences between muscle groups or between the same muscle in each arm (*p < 0.05, **p < 0.01, and ***p < 0.001). BIC, biceps brachii; BRD, brachioradialis; D, dominant; HCP, hemiparetic cerebral palsy; ND, non-dominant; NP, non-paretic; P, paretic; TD, typical development; TRI, lateral head of the triceps brachii; WFL, extrinsic wrist/finger flexors (flexor carpi radialis and flexor digitorum profundus); WEX, extrinsic wrist/finger extensors (extensor carpi radialis, extensor digitorum communis).

When comparing mirroring between arms for the TD group (Figure 4A), there was a significant main effect of muscle [*F*_(__5_, _96__)_ = 7.340, *p* < 0.001]. *Post hoc* pairwise comparisons between muscles revealed a significant difference in mirrored ratios between BIC and TRI (higher in TRI; *t*_96_ = −3.381, *p* = 0.011, *g* = −0.103), BIC and IDL (higher in IDL; *t*_96_ = −4.926, *p* < 0.001, *g* = −0.123), BIC and WEX (higher in WEX; *t*_96_ = −3.640, *p* = 0.005, *g* = −0.076), IDL and WFL (higher in IDL; *t*_96_ = 3.705, *p* = 0.001, *g* = 0.136), and IDL and BRD (higher in IDL; *t*_96_ = 3.705, *p* = 0.005, *g* = 0.091). Other tested effects were not significant {test arm [*F*_(__1_, _96__)_ = 1.555, *p* = 0.215], test arm by muscle [*F*_(__5_, _96__)_ = 0.965, *p* = 0.443]}. When comparing mirroring between arms for the HCP group, there was a significant main effect of test arm [*F*_(__1_, _156__)_ = 83.543, *p* < 0.001], muscle [*F*_(__5_, _156__)_ = 4.213, *p* = 0.001], and the interaction of test arm by muscle [*F*_(__5_, _126__)_ = 3.793, *p* = 0.003] demonstrating a significant directionality of mirroring from the NP arm to the paretic arm. *Post hoc* pairwise comparisons revealed a significant difference in mirrored ratios between BIC and WEX (higher in WEX; *t*_156_ = −3.097, *p* = 0.030, *g* = 0.008), TRI and WEX (higher in WEX; *t*_156_ = −3.774, *p* = 0.003, *g* = 0.001), and BRD and WEX (higher in WEX; *t*_156_ = −3.544, *p* = 0.007, *g* = 0.003). Additionally, the mirrored ratio was significantly higher when the NP arm was contracting all muscles except WEX (*t*_156_ = 1.819, *p* = 0.071) according to *post hoc* pairwise comparisons (BIC: *t*_156_ = 6.437, *p* < 0.001, *g* = 2.077; TRI: *t*_156_ = 3.418, *p* = 0.001, *g* = 0.961; IDL: *t*_156_ = 2.139, *p* = 0.034, *g* = 0.768; WFL: *t*_156_ = 2.750, *p* = 0.007, *g* = 0.805; BRD: *t*_156_ = 5.826, *p* < 0.001, *g* = 1.433) as shown in Figure 4B. In summary, the HCP group demonstrated more mirrored muscle activation when contracting the NP arm in all muscles except the WEX whereas the TD group demonstrated small mirrored ratios in each arm for all muscles.

### Bilateral Activation During Submaximal Reaching Tasks

Multiple combinations of muscle activations can be employed to maximize the torque production in one direction. Therefore, evaluating muscle activations during a dual task at multiple joints provided an avenue to explore mirrored activation in a specific task at submaximal effort levels. Total arm muscle activity increased with shoulder abduction load level for all arms tested (Table 2). The total normalized paretic arm EMG in those with HCP was higher than the ND arm of those with TD indicating greater relative activity in those with HCP.

**TABLE 2:** Mean (SD) total reaching arm electromyography (EMG) sums per load level.

	**Reaching arm**			
	**Dominant/non-paretic**		**Non-dominant/paretic**	
**Timepoint 1**	**TD (*n* = 9)**	**HCP (*n* = 14)**	**TD (*n* = 9)**	**HCP (*n* = 14)**
Table	0.214 (0.13)	0.211 (0.20)	0.213 (0.10)	0.393 (0.19)
20% MVT	0.291 (0.12)	0.262 (0.16)	0.279 (0.08)	0.520 (0.22)
35% MVT	0.397 (0.14)	0.372 (0.17)	0.424 (0.10)	0.596 (0.24)
50% MVT	0.568 (0.15)	0.529 (0.32)	0.669 (0.15)	0.744 (0.35)
65% MVT	0.838 (0.27)	0.653 (0.31)	0.907 (0.15)	0.922 (0.38)
80% MVT	1.058 (0.34)	0.899 (0.44)	1.140 (0.14)	1.203 (0.51)

**Timepoint 2**	**TD (*n* = 9)**	**HCP (*n* = 14)**	**TD (*n* = 9)**	**HCP (*n* = 14)**

Table	0.848 (0.45)	0.723 (0.34)	0.784 (0.20)	1.279 (0.44)
20% MVT	0.877 (0.53)	0.780 (0.45)	0.982 (0.23)	1.333 (0.56)
35% MVT	0.874 (0.36)	0.982 (0.56)	1.193 (0.27)	1.555 (0.53)
50% MVT	1.186 (0.56)	1.076 (0.45)	1.372 (0.33)	1.616 (0.68)
65% MVT	1.435 (0.57)	1.391 (0.59)	1.576 (0.29)	1.710 (0.59)
80% MVT	1.601 (0.61)	1.355 (0.54)	1.687 (0.31)	1.818 (0.64)

**Timepoint 3**	**TD (*n* = 9)**	**HCP (*n* = 14)**	**TD (*n* = 9)**	**HCP (*n* = 14)**

Table	1.116 (0.62)	0.833 (0.51)	1.144 (0.21)	1.211 (0.47)
20% MVT	1.172 (0.64)	0.940 (0.62)	1.384 (0.41)	1.294 (0.57)
35% MVT	1.201 (0.52)	1.214 (0.72)	1.551 (0.23)	1.484 (0.45)
50% MVT	1.555 (0.72)	1.280 (0.64)	1.856 (0.54)	1.567 (0.56)
65% MVT	1.742 (0.67)	1.533 (0.69)	1.849 (0.27)	1.733 (0.56)
80% MVT	1.827 (0.67)	1.484 (0.69)	1.911 (0.43)	1.886 (0.72)

*Mean (SD) normalized EMG summation for the reaching arm where a value of 6 indicates that all muscles are contracting at 100% (maximum possible EMG summation value). HCP, hemiparetic cerebral palsy; MVT, maximum voluntary torque; SD, standard deviation; TD, typical development.*

**FIGURE 5:**
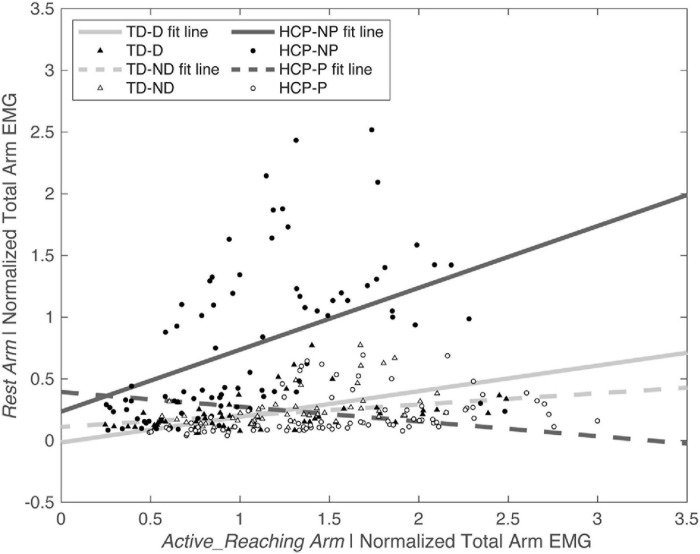
Normalized *Active_Reaching Arm* EMG vs. *Rest Arm* EMG at timepoint 2 during the acceleration phase of the reach. Equations from linear model: TD-D: *Rest Arm* = 0.11109 + 0.090719 × *Active_Reaching Arm* EMG; TD-ND: *Rest Arm* = –0.014667 + 0.20751 × *Active_Reaching Arm* EMG; HCP-NP: Rest Arm = 0.23554 + 0.50113 × *Active_Reaching Arm* EMG; HCP-P: *Rest Arm* = 0.3936 + –0.11923 × *Active_Reaching Arm* EMG. D, dominant; HCP, hemiparetic cerebral palsy; ND, non-dominant; NP, non-paretic; P, paretic; TD, typical development.

There was a significant main effect of *Active_ReachingArm* EMG on *Rest Arm* EMG [*F*_(__1_, _266__)_ = 21.541, *p* < 0.001] and the interaction of group by *Active_ReachingArm* EMG [*F*_(__1_, _258__)_ = 24.317, *p* < 0.001]. This significant interaction highlights that the slopes of the co-activation relationship between the *Active_Reaching Arm* and the *Rest Arm* were different depending on which arm was reaching (Figure 5). In the HCP group at timepoint 2, the relationship between *Active_Reaching Arm* EMG and *Rest Arm* EMG has a steeper slope when reaching with the NP arm compared to reaching with the paretic arm (*t*_257_ = 8.425, *p* < 0.001). In the TD group, there was not a significant difference in the *Active_Reaching Arm* EMG vs. *Rest Arm* EMG relationship between reaching with the dominant arm or ND arm at timepoint 2 (*t*_247_ = 1.158, *p* = 0.248). This result highlights that the *Rest Arm* EMG increases more proportionally to the increase in *Active_Reaching Arm* EMG in the HCP group when reaching with the NP arm but there is not a difference between arms for the TD group. Additional slopes and statistical comparisons from timepoints 1 and 3 are shown in Tables 3, 4 and demonstrate similar results in the HCP group.

**TABLE 3:** Linear model of *Active_Reaching Arm* EMG and *Rest Arm* EMG.

	**TD-D (*n* = 9)**	**TD-ND (*n* = 9)**	**HCP-NP (*n* = 14)**	**HCP-P (*n* = 14)**
**Timepoint 1**				
Y-intercept	0.069	0.065	0.070	0.033
Slope	0.111	0.138	0.637	0.182
Slope 95% CI	[0.020, 0.203]	[0.047, 0.229]	[0.560, 0.714]	[0.115, 0.250]
**Timepoint 2**				
Y-intercept	0.111	−0.015	0.236	0.394
Slope	0.091	0.208	0.501	−0.119
Slope 95% CI	[−0.044, 0.226]	[0.036, 0.379]	[0.391, 0.611]	[−0.228, −0.009]
**Timepoint 3**				
Y-intercept	0.134	−0.029	0.176	0.371
Slope	0.103	0.211	0.540	−0.084
Slope 95% CI	[−0.022, 0.228]	[0.039, 0.385]	[0.441, 0.638]	[−0.198, 0.030]

*CI, confidence interval; D, dominant; HCP, hemiparetic cerebral palsy; ND, non-dominant; NP, non-paretic; P, paretic; TD, typical development.*

**TABLE 4:** Group comparison of slopes from linear models.

		**Timepoint 1**		**Timepoint 2**		**Timepoint 3**	
		***t***	***p***	***t***	***p***	***t***	***p***
TD-D	TD-ND	0.404	0.686	1.158	0.248	1.084	0.279
TD-D	HCP-NP	8.640	< 0.001	4.634	< 0.001	5.398	< 0.001
TD-D	HCP-P	1.232	0.219	2.375	0.018	2.176	0.030
HCP-NP	HCP-P	9.221	< 0.001	8.425	< 0.001	8.573	< 0.001
HCP-NP	TD-ND	8.219	< 0.001	2.837	0.005	3.246	< 0.001
HCP-P	TD-ND	0.772	0.441	3.160	0.002	2.815	0.005

*The slopes of the model fits for *Active_Reaching Arm* EMG vs. *Rest Arm* EMG were compared between arms and groups. At timepoint 1, the slope of the HCP-NP fit line was significantly different from the slopes of the other three lines. However, the slopes of the TD-D, TD-ND, and HCP-P arms when compared were not significantly different from each other. At timepoint 2, all slopes were significantly different between groups and arms except the TD-D compared to the TD-ND. Finally, at timepoint 3, all slopes were significantly different from each other except the TD-D/TD-ND. D, dominant; HCP, hemiparetic cerebral palsy; ND, non-dominant; NP, non-paretic; P, paretic; TD, typical development.*

## Discussion

This study investigated the presence/absence and directionality of mirrored muscle activation in the whole upper limb of young people with and without hemiplegia during static and dynamic upper limb tasks. Group results of the experimental tasks were evaluated for one of four possible mirroring combinations: paretic arm mirrors (P_Mirror), NP arm mirrors (N_Mirror), both arms mirror (B_Mirror), and neither arm mirrors (X_Mirror). In the static tasks, mirrored activation ratios were larger in individuals with HCP compared to the TD group when contracting the NP arm but not when contracting the paretic arm. In dynamic conditions, individuals with HCP showed significantly greater interlimb co-activation when reaching with the NP arm compared to reaching with the paretic arm. These results show a clear difference in the control of the paretic vs. the NP arm, providing insight into the underlying neural mechanisms that may be involved in arm control after early brain lesions. The TD group showed minimal mirrored activity during static and dynamic tasks. Of the four mirroring scenarios evaluated, the results of both static and dynamic tasks align most closely with the P_Mirror hypothesis describing a unidirectional mirroring that is driven by the activation of the NP arm.

### Directionality of Mirroring

Electromyography (EMG) quantification offers higher resolution than is afforded by observational rating scales alone to provide insight into how the nervous system may be activating both arms simultaneously (Green, 1967; Carr et al., 1993; Carr, 1996). Furthermore, the use of the behavioral tasks of volitional contractions and movement goes beyond probing connectivity that is elicited by TMS and enables quantifying the capability of the nervous system to engage the available descending motor pathways. Green evaluated bilateral motor unit action potentials during single joint movements at the elbow wrist, and fingers (Green, 1967). The key finding of this study was that associated movements occurred only on the paretic side when the NP side was being activated (Green, 1967). Our cohort demonstrates a similar unidirectionality in mirrored activations. We found large mirrored ratios and an increase in *Rest Arm* EMG as a function of *Active_Reaching Arm* EMG when the NP arm, but not the paretic arm, was the active arm suggesting unidirectional mirroring. Specifically, there is activation in the paretic arm when the NP arm is leading the contraction or movement whereas there is minimal activation in the NP arm when the paretic arm is leading. This direction of mirroring suggests activation of ipsilaterally terminating CST projections from the non-lesioned hemisphere (Staudt et al., 2004). In the cohort studied by Staudt et al. (2004) mirroring that occurred in the paretic limb when activating the NP limb was only seen in those with ipsilateral connectivity as detected by TMS, linking this directionality of mirroring to ipsilateral fast conducting CST pathways. In contrast, mirroring in the NP arm when leading the movement with the paretic limb is more likely a non-specific overflow seen in individuals with maintained contralateral CST from the lesioned hemisphere (Staudt et al., 2004). The directionality of mirroring found in our results could indicate ipsilaterally originating pathways as the neural mechanism of the P_Mirror hypothesis. Additional investigation with TMS in our cohort would be pertinent to confirm the cortical origins of the mirrored activations we are measuring peripherally.

Some studies have shown the opposite directionality of mirror movements (Klingels et al., 2016; Riddell et al., 2019). Riddell et al. (2019) assessed the directionality of mirror movements by aggregating the results of clinical scores from finger tapping, sequencing, and grasp tasks and TMS measures of CST organization in a cohort of participants with HCP. A key finding was that the population with ipsilateral CST arrangement showed a stronger directionality in mirroring compared to those with non-ipsilateral CST arrangements. The directionality found for those with ipsilateral CST arrangement was primarily mirroring in the NP arm during focused fine motor tasks in the paretic hand which is opposite of the results in the present study as well as previous studies (Green, 1967; Staudt et al., 2004). There is still more to be understood regarding the extent to which the directionality of mirroring is inherent in the connectivity of the CST and how much is driven by a participant employed strategy to activate the NP hand to complete the task in the paretic hand. Our methodology without feedback on muscle activation levels may be advantageous in reducing the use of a strategy to achieve a specific outcome.

### Proximal vs. Distal Muscles

Characterization of the whole limb as well as exploring the directionality of between limb muscle co-activation enables a more complete interpretation of the neural mechanisms implicated in mirror movements after early brain injury. A key focus of previous mirror movement studies has been at the hand demonstrating stronger mirroring primarily from the paretic hand to the NP hand (Woods and Teuber, 1978; Kuhtz-Buschbeck et al., 2000; Riddell et al., 2019), the opposite directionality seen in our dataset of the whole limb. A study that included the elbow found directionality primarily from the NP arm to the paretic arm (Green, 1967) which is similar to our results for the BIC, BRD, and TRI during the isometric maximum contractions. Sukal-Moulton et al. (2013) systematically investigated the effect of maximal and submaximal isometric and isokinetic efforts of the NP elbow on involuntary isometric torque production by the paretic elbow. They found that mirroring was more prevalent in individuals with lesions sustained under 6 months of age compared to those with later lesion timings and that this earlier injury timing group was less able to suppress elbow flexion mirroring into the paretic arm (Sukal-Moulton et al., 2013). While the focus of our study was on the spontaneous activity of the *Rest Arm* without instructions to suppress any activation, we demonstrate increased paretic arm EMG activity with load level during reaching with the NP arm which is similar to the increased paretic arm torque with NP arm effort level found previously (Sukal-Moulton et al., 2013). Interestingly, we found that while there was clear directionality in all of the proximal muscles tested, the WEX showed similar magnitude of mirroring irrespective of which arm was the *Active_Isometric Arm*. The similarity in mirroring between hands for this distal muscle highlights a potential difference in descending control to the hand. The necessity of the hand for fine motor tasks underscores the need for further investigation into the independent control of the hand.

### Neural Mechanisms Underlying Mirror Movements

The presence of mirrored muscle activation during NP arm contraction found in the HCP group of our study would suggest the hypothesized of maintenance of simultaneous control of both arms from one hemisphere after early lesions to the brain. Possible mechanisms of descending pathway reorganization after a unilateral lesion have been explored in previous studies. Staudt (2010) suggests that the interruption of the typical pruning process of the CST after a unilateral lesion enables the non-lesioned hemisphere to maintain direct CST projections to motor neuron pools innervating both arms. This mechanism could coincide with the branched projection hypothesis put forth by Carr (1996) and could be the underlying mechanism in our key findings. Hawe et al. (2013) suggest instead that the integrity and volume of the white matter in the corpus callosum may be implicated in the inability to suppress mirror movement in individuals with early unilateral lesions. In a comparative literature review evaluating the ipsilateral CST projection from the non-lesioned hemisphere hypothesis against the insufficient interhemispheric inhibition hypothesis, Kuo et al. (2018) assert that the presence of mirror movements is most indicative of an ipsilateral CST reorganization. Based on the directionality of mirroring that we measured in our cohort, we also postulate that the presence of these mirrored activations is likely due to maintained ipsilaterally terminating CST projections from the non-lesioned hemisphere. In contrast to maintained ipsilateral CST projections for upper limb control, the corticoreticulospinal tract is implicated in the abnormal flexion synergies of the upper extremity in adults after stroke (Schwerin et al., 2008; McPherson et al., 2018). Pronounced flexion synergies driven by shoulder abduction during reaching are seen in adult-onset hemiplegia, whereas our previous work in pediatric-onset hemiplegia largely showed an absence of this synergy pattern in those with early lesions while completing a ballistic reaching task (Hill and Dewald, 2020). This provides evidence against the activation of brainstem mediated pathways during volitional movements and would support the hypothesis that mirrored activation could be driven by ipsilateral CST connections. Interestingly, our results show greater total arm activation in the paretic arm of those with HCP compared to the ND arm of those with TD (Table 2). Sukal-Moulton et al. (2014) detected an overflow in within limb activation during distal joint MVTs in those with earlier injuries. Specifically, they demonstrated significant coupling between elbow flexion and wrist/finger flexion compared to those without neurological impairment pointing out within limb coupling that is distinct from the proximally driven flexion synergy pattern seen in adults post stroke.

### Limitations

We collected data for only six muscles due to setup time and surface area available on participants’ arms, however, this snapshot did include at least one muscle crossing each joint of the upper limb. While we cued participants to relax the *Rest Arm* during active arm efforts, we did not provide feedback that would encourage suppression of mirrored activations as that was out of the scope of this particular study. In this study we have recorded solely muscle activation from the periphery and have not taken any measurements directly from the cortex. In order to confirm the cortical component at the origin of mirrored muscle activations, neuroimaging such as TMS, fMRI, or calculating EEG/EMG wavelet coherence (Xi et al., 2020) would be required in combination with our peripheral measures.

### Clinical Implications

Tasks of daily life require the ability to independently control joints within a limb as well as the coordinated control of the hands in concert. Bimanual tasks often require one arm/hand to stabilize and the other to engage in manipulation (Johansson et al., 2006; Wang and Sainburg, 2007). Individuals with HCP frequently have difficulty with unimanual tasks involving the paretic hand and bimanual tasks involving both hands (Holmefur et al., 2010). The presence of an alternative wiring pattern of the CST may be responsible for the persistence of mirror movements seen in this population, and is unlikely to be reduced with the use of unilateral training. The use of EMG could offer an additional measurement of neural output that could be incorporated into interventions purposed to decrease mirrored movements and improve function. Unlike other neuroimaging approaches which are more complex and possibly contraindicated for this population, EMG recording can be used in a clinical setting alongside of other clinical assessments to investigate with finer resolution where the issues of co-activation are present in a similar manner as the use of the Windmill-task (Zielinski et al., 2018). EMG also has the potential to facilitate more rapid diagnosis of CST connectivity that could help guide the development of interventions though additional investigation would be needed for validation with the gold standard of TMS.

## Data Availability Statement

The raw data supporting the conclusions of this article will be made available by the authors, without undue reservation.

## Ethics Statement

The studies involving human participants were reviewed and approved by the Northwestern University Institutional Review Board. Written informed consent to participate in this study was provided by the participants’ legal guardian/next of kin.

## Author Contributions

NH and JD contributed to the conception and design of the work. NH contributed to the data acquisition, data analysis, and initial manuscript preparation. All authors contributed to the interpretation of the data, wrote and edited the manuscript, approved the submitted version, and agreed to be accountable for all aspects of the work.

## Conflict of Interest

The authors declare that the research was conducted in the absence of any commercial or financial relationships that could be construed as a potential conflict of interest.

## Publisher’s Note

All claims expressed in this article are solely those of the authors and do not necessarily represent those of their affiliated organizations, or those of the publisher, the editors and the reviewers. Any product that may be evaluated in this article, or claim that may be made by its manufacturer, is not guaranteed or endorsed by the publisher.
